# Mini Review: Anticholinergic Activity as a Behavioral Pathology of Lewy Body Disease and Proposal of the Concept of “Anticholinergic Spectrum Disorders”

**DOI:** 10.1155/2016/5380202

**Published:** 2016-09-22

**Authors:** Koji Hori, Kimiko Konishi, Misa Hosoi, Hiroi Tomioka, Masayuki Tani, Yuka Kitajima, Mitsugu Hachisu

**Affiliations:** ^1^Department of Neuropsychiatry, St. Marianna University, School of Medicine, Kanagawa, Japan; ^2^Department of Psychiatry, Showa University Northern Yokohama Hospital, Kanagawa, Japan; ^3^Tokyo Metropolitan Tobu Medical Center for Persons with Developmental/Multiple Disabilities, Tokyo, Japan; ^4^Department of Psychiatry, Showa University East Hospital, Tokyo, Japan; ^5^Department of Anesthesiology, School of Medicine, Juntendo University, Tokyo, Japan; ^6^Department of Pharmaceutical Therapeutics, Division of Clinical Pharmacy, School of Pharmacy, Showa University, Tokyo, Japan

## Abstract

Given the relationship between anticholinergic activity (AA) and Alzheimer's disease (AD), we rereview our hypothesis of the endogenous appearance of AA in AD. Briefly, because acetylcholine (ACh) regulates not only cognitive function but also the inflammatory system, when ACh downregulation reaches a critical level, inflammation increases, triggering the appearance of cytokines with AA. Moreover, based on a case report of a patient with mild AD and slightly deteriorated ACh, we also speculate that AA can appear endogenously in Lewy body disease due to the dual action of the downregulation of ACh and hyperactivity of the hypothalamic-pituitary-adrenal axis. Based on these hypotheses, we consider AA to be a behavioral pathology of Lewy body disease. We also propose the concept of “anticholinergic spectrum disorders,” which encompass a variety of conditions, including AD, Lewy body disease, and delirium. Finally, we suggest the prescription of cholinesterase inhibitors to patients in this spectrum of disorders to abolish AA by upregulating ACh.

## 1. Introduction

The neurotransmitter acetylcholine (ACh) and anticholinergic activity (AA) can bind to the muscarinic acetylcholine receptor [1]. AA includes every substance that binds the muscarinic acetylcholine receptor; however, generally speaking AA antagonizes ACh to the muscarinic acetylcholine receptor. Therefore, AA disturbed the function of ACh. AA predominantly disturbs memory function [2, 3], with psychotic symptoms more prominent than cognitive dysfunctions [4, 5]. The main cause of AA is prescribed medication [6], but physical illness [7] and mental stress which cause elevated cortisol [8] also cause AA. We previously reported that AA was also caused by ACh downregulation and proposed our hypothesis of the endogenous appearance of AA in Alzheimer's disease (AD) [9, 10]. That is, because ACh regulates not only cognitive function but also the inflammatory system, when ACh downregulation reaches a critical level, inflammation is increased, triggering the appearance of cytokines with AA. These processes might also accelerate AD pathologies [9, 10]. Moreover, we speculated that, in addition to AD, other neurocognitive disorders such as Lewy body disease (LBD) and delirium are related to AA and that the appearance of AA in LBD is also related to an endogenous factor [11]. In this article, we rereview our hypotheses surrounding the endogenous appearance of AA in AD [9, 10] and LBD [11]. We also theorize that the onset of clinical symptoms in LBD depends on the endogenous appearance of AA in LBD and propose a new concept of “anticholinergic spectrum disorders.”

## 2. Hypothesis of Endogenous Anticholinergic Activity in Alzheimer's Disease

We previously evaluated the relationship between clinical symptoms and AA in 76 AD patients [12]. Serum anticholinergic activity (SAA), a peripheral marker of anticholinergic burden, was positive in 26 of these patients; the other 50 were negative for SAA. Delusions, hallucinations, diurnal rhythm disturbances, and global cognitive dysfunctions were significantly more severe in the SAA-positive group than in the SAA-negative group, and the patients in the SAA-positive group took more psychotropic medicines. Moreover, SAA positivity was more related to psychotic symptoms than cognitive dysfunctions. Our results indicated that clinical symptoms and prescription of psychotropic medicines are factors related to SAA, particularly psychotic symptoms. Because these results were from a cross-sectional study, we could not elucidate the causal relationships among these three factors (psychotic symptoms, the prescription of psychotropic medicines, and SAA). However, we speculated that there might be a cyclic relationship among the factors. Accordingly, we named this association the “vicious cycle of anticholinergic activity in AD (VCAA)” [12].

Positive SAA can be caused by various medicines and AA worsens clinical psychiatric symptoms. However, psychotropic medicines are generally prescribed for the clinical psychiatric symptoms of agitation and psychosis in AD [13, 14]. Therefore, the relationship among prescribed psychotropic medicines, SAA, and clinical symptoms—especially hallucinations and a disturbed diurnal rhythm—might be cyclic. Moreover, because psychotropic medicines are typically prescribed for the clinical psychiatric symptoms of agitation and psychosis in AD [13, 14], psychotropic medicines might not be the first step in this cycle. There might be a causal relationship between the other two factors comprising the VCAA, namely, psychotic symptoms and the use of psychotropic medicines, with AA worsening psychiatric symptoms. Therefore, we consider the appearance of AA to be the first step in the VCAA: that is, AA appears endogenously in AD [12]. In fact, there is a high probability that the pathogenesis of AD involves neuronal degeneration due to oxidative stress, and it has been shown that amyloid might be able to generate free radicals [15]. On the other hand, an endogenous ligand of the muscarinic receptor is found to a greater extent in the AD brain than in the nondemented control brain, and the endogenous ligand of the muscarinic receptor seems to be a low-molecular weight substance of 100–1,000 Da that is catalyzed by oxidation [16]. Thus, SAA might not always derive purely from prescribed psychotropic medicines; it may also derive from endogenous oxidative products [12].

In short, we hypothesized that the relationship among psychotic symptoms, positive SAA, and the prescription of psychotropic medicines is a cyclic one and that AA appears endogenously in AD [12], because psychotropic medicines are prescribed for psychotic symptoms [13, 14]. We also speculated about the mechanism behind the endogenous appearance of AA in AD [9, 10]. ACh regulates not only cognitive function but also the inflammatory system [17, 18]. Therefore, we surmised that because AD is characterized by the downregulation of ACh, the inflammatory system is upregulated in AD when the level of ACh reaches a specific threshold (i.e., moderately severe disease) [9, 10]. Thus, downregulation of ACh would cause downregulation of the anti-inflammatory pathway (the cholinergic anti-inflammatory pathway), permitting upregulation of the inflammatory pathway [17, 18]. The hyperactive inflammation generates cytokines with AA, such as C-reactive protein [19].

Moreover, stimulation of muscarine 1 receptor is favorable for reducing amyloid pathology [20, 21]. Therefore, we considered that AA accelerates amyloid pathology. In fact, anticholinergic loads are reported to increase amyloid pathology [22, 23]. We thus proposed the hypothesis of the endogenous appearance of AA in AD.

## 3. Dual Actions of Anticholinergic Inserts Cause Anticholinergic Activity and the Endogenous Appearance of Anticholinergic Activity in Lewy Body Disease

We then encountered a 74-year-old woman with positive SAA, although her cognitive decline was not at a sufficiently critical level to elicit endogenous AA [24]. In this instance, we hypothesized that the SAA positivity was induced by the addition of mental stress to a preexisting ACh downregulation [24]. We consider mental stress and other factors besides ACh downregulation to be capable of inducing SAA, to be so-called “AA inserts.” In this context, because there are other AA inserts besides ACh downregulation [9, 10], such as the prescribed medication [6], physical illness [7], and mental stress [8], dual actions of AA inserts can also cause AA when the ACh level does not reach a critical level (i.e., at the stage of mild cognitive impairment or at a mild stage of the disease) [24]. Based on this case, we speculated that the appearance of AA might cause psychiatric symptoms such as delusions, hallucinations, and diurnal rhythm disturbances in delirium and LBD based on the dual actions of ACh downregulation [11] (if ACh is not deteriorated or overloaded, the intact ACh system can be upregulated and compensate for another AA insert [10]) and the effects of another AA insert.

We also previously evaluated the relationship between postoperative delirium and SAA [11]. Although delirium is considered an important issue among elderly patients in various settings, the mechanism underlying delirium is poorly understood [25]. We concluded that delirious patients fail to compensate for the increase in AA, making it important to pay close attention to the perioperative transition of the SAA level in relation to delirium, rather than focusing on a single SAA level [11].

The factor suggesting the endogenous appearance of AA in LBD is the dual action of a deteriorated autonomic parasympathetic nervous system and a relatively minor decrease in ACh [11]. This is attributable to the induction of AA in LBD, with the deteriorated autonomic parasympathetic nervous system increasing the activity of the hypothalamic-pituitary-adrenal (HPA) axis [26] and hypercortisolism [27, 28]. If inflammatory processes are caused by AA, it is plausible to conclude that corticosteroids would inhibit the action of AA because of their anti-inflammatory properties.

Nonetheless, corticosteroids have been reported to induce or increase AA in the brain [8] and cause delirium [25]. Typically, the corticosteroid level in plasma is high early in the morning and rapidly declines thereafter. We theorized that this rapid reduction might cause immune system disinhibition, with immune system activation in the afternoon, evening, and night [10], because cortisol levels after awakenings, such as in the morning, might be affected in neurocognitive and neuropsychiatric disorders [10]. If the blood level of corticosteroids rises above normal, the subsequent decline in the level can be expected to be greater, where it is expected that the inflammatory state activated the AA. Therefore, it seems logical that even if AA does not appear early in the morning, it may appear by noon or later. This mechanism might explain why patients with delirium can appear calm in the morning but delirious in the late afternoon and at night (i.e., sundowning) [10]. We also believe that deteriorated parasympathetic autonomic nervous function causes AA in patients with LBD based on the small degree of downregulation of ACh and that AA appears endogenously in LBD [11].

Briefly, AA appears earlier in LBD than in AD because of the combination of HPA axis hyperactivity and the small degree of ACh downregulation. In delirium, AA appears, as in LBD, due to the combination of these two factors. In LBD, HPA axis hyperactivity occurs endogenously by way of dysfunction of the parasympathetic nervous system [27, 28]. In contrast, HPA axis hyperactivity occurs exogenously in delirium due to mental stress and/or physical illnesses. AA is thus related to the pathogenesis of AD, LBD, and delirium.

**Figure 1:**
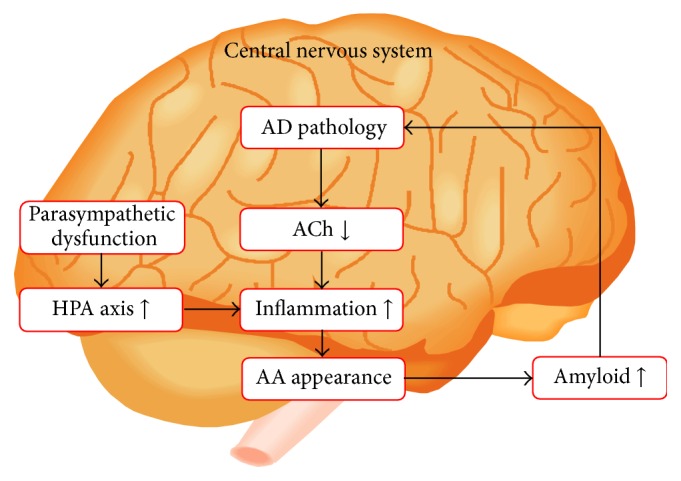
AA in LBD appears earlier than in Alzheimer's disease because of a combination of HPA axis hyperactivity and a small degree of ACh downregulation. In LBD, HPA axis hyperactivity occurs endogenously due to dysfunction of the parasympathetic nervous system. We refer to these processes as the hypothesis of the endogenous appearance of AA in LBD. Based on the small degree of ACh downregulation, HPA axis hyperactivity caused by deterioration of the autonomic parasympathetic nervous system gradually worsens before finally inducing hyperactive inflammation, which also causes AA. Continuous and recurrent appearance of AA exacerbates the amyloid pathology and further downregulates ACh. The order of the process is as follows: (1) deterioration of the parasympathetic nervous system, (2) hyperactivity of the HPA axis, (3) appearance of AA, and (4) downregulation of ACh. AA: anticholinergic activity, ACh: acetylcholine, AD: Alzheimer's disease, HPA axis: hypothalamic-pituitary-adrenal axis, and LBD: Lewy body disease. This figure is reproduced from Hori et al. [9] with permission from the Japanese Society of Neuropsychopharmacology (Tokyo, Japan).

We can explain the onset of clinical symptoms using our hypothesis of the endogenous appearance of AA in LBD. This hypothesis is shown in Figure 1. Briefly, based on a small degree of ACh downregulation, HPA axis hyperactivity caused by a deteriorated autonomic parasympathetic nervous system gradually worsens and finally induces hyperactive inflammation, which also causes AA. Continuous and recurrent appearance of AA exacerbates the amyloid pathology and further downregulates ACh. We divided this entire process into the following three stages, in this order: (1) deterioration of the parasympathetic nervous system (and hyperactivity of the HPA axis); (2) appearance of AA; and (3) downregulation of ACh caused by continuous and recurrent appearance of AA.

**Figure 2:**
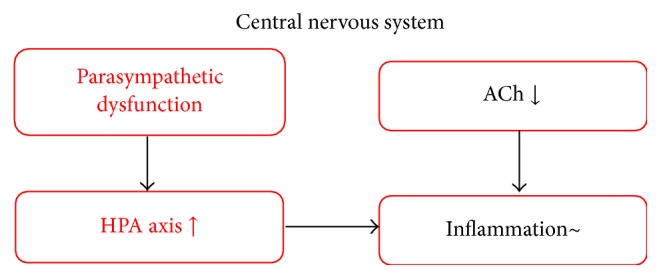
At the stage of parasympathetic nervous system deterioration, which corresponds to the prodromal stage of LBD, HPA axis hyperactivity gradually worsens but does not reach the level at which AA is induced. Therefore, symptoms related to dysfunction of the parasympathetic nervous system occur in LBD, such as REM behavioral symptoms, syncope, and constipation. Moreover, depression, which is related to HPA axis hyperactivity, also develops.

At the stage of parasympathetic nervous system deterioration, which corresponds to the prodromal stage of LBD, HPA axis hyperactivity gradually worsens but does not reach the level at which AA is induced. Therefore, symptoms related to dysfunction of the parasympathetic nervous system occur in LBD, such as REM behavioral symptoms, syncope, and constipation [29]. Moreover, depression, which is related to HPA axis hyperactivity, also develops (Figure 2). We have already reported that anxiety and affective disturbances in AD patients are connected to delusion, hallucination, and aggressiveness by aging and the disease progress [30]. Therefore, if the degree of ACh dysfunction is relatively large, depression appears as behavioral and psychological symptoms of dementia in AD.

**Figure 3:**
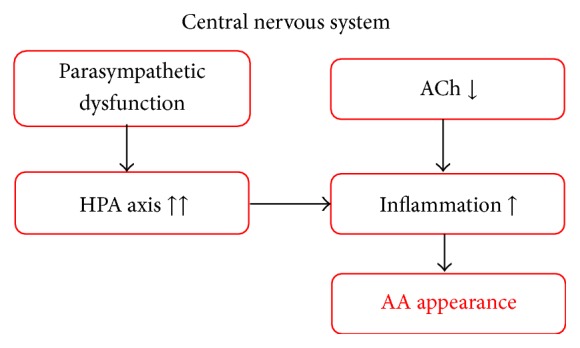
At the stage of AA appearance, which corresponds to the early stage of LBD, HPA axis hyperactivity reaches a critical level and induces AA. In the early stage of LBD, symptoms related to AA develop, including psychotic symptoms such as visual hallucinations, delusions, and diurnal rhythm disturbances. These symptoms are similar to those of delirium and are included as the core symptoms of the diagnostic criteria.

At the stage of AA appearance, which corresponds to the early stage of LBD, HPA axis hyperactivity reaches a critical level and induces AA. In the early stage of LBD, symptoms related to AA develop, including psychotic symptoms such as visual hallucinations, delusions, and diurnal rhythm disturbances. These symptoms are similar to those of delirium and are included as the core symptoms of the diagnostic criteria [29] (Figure 3). When patients show these psychotic symptoms, they are typically referred to a department of psychiatry. Similarly, patients with LBD are also generally referred to the same department at this stage, and we consider AA (psychosis) and HPA axis hyperactivity (depression), as triggers of AA, to be behavioral pathologies of LBD.

**Figure 4:**
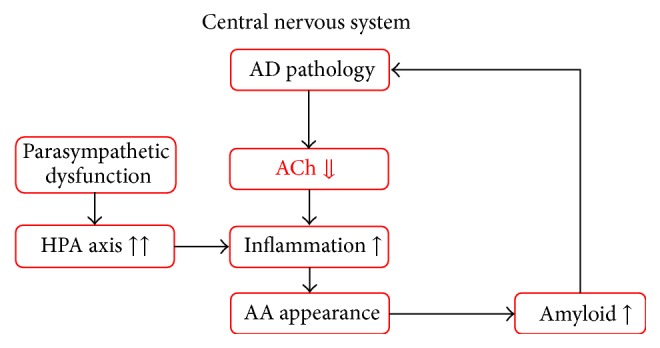
At the stage of ACh downregulation, corresponding to the late stage of LBD, the patient develops symptoms related to ACh downregulation, which is induced by the continuous and recurrent appearance of AA. These symptoms include memory disturbances and disorientation to time and place. AA: anticholinergic activity, ACh: acetylcholine, AD: Alzheimer's disease, HPA axis: hypothalamic-pituitary-adrenal axis, and LBD: Lewy body disease.

At the stage of ACh downregulation, corresponding to the late stage of LBD, the patient develops symptoms related to ACh downregulation, which is induced by the continuous and recurrent appearance of AA. These symptoms include memory disturbances and executive dysfunction [31] (Figure 4).

AA appears when the ACh deterioration reaches a critical level, that is, at a moderate stage in AD. Therefore, AA is related not only to behavioral symptoms, such as delusions, hallucinations, and diurnal rhythm disturbances [12], but also to cognitive dysfunctions, such as memory disturbances and executive dysfunction. However, the ACh deterioration in LBD and delirium is not as severe as that of AD when AA appears. Therefore, only behavioral symptoms are prominent in LBD and delirium, such as delusions, hallucinations, and diurnal rhythm disturbances. This is why the symptoms of LBD and delirium are similar. We consider the pathophysiology of the clinical symptoms of LBD to be related to AA.

## 4. Proposal of “Anticholinergic Spectrum Disorders” in Neurocognitive Disorders

**Figure 5:**
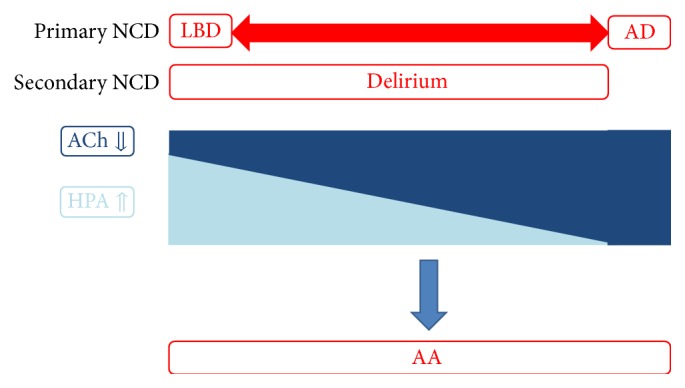
When the ACh deterioration is small, a larger AA insert of HPA axis dysfunction is necessary for the appearance of AA. However, when the ACh deterioration is large, a small AA insert of HPA axis dysfunction is sufficient for the appearance of AA. Of course, when the ACh deterioration is at a critical level, AA appears without HPA axis dysfunction. However, if ACh is not deteriorated or overloaded, the intact ACh system can be upregulated and compensate for any other AA inserts. We refer to the conditions encompassed by this concept as “anticholinergic spectrum disorders,” which include AD, LBD, and delirium. AD and LBD are primary NCDs, whereas delirium is a secondary NCD. AA: anticholinergic activity, ACh: acetylcholine, AD: Alzheimer's disease, HPA axis: hypothalamic-pituitary-adrenal axis, LBD: Lewy body disease, and NCD: neurocognitive disorder.

Based on these hypotheses, we propose that certain neurocognitive disorders, such as AD, LBD, and delirium, be considered “anticholinergic spectrum disorders.” In AD, AA appears endogenously when the downregulation of ACh reaches a critical level. In contrast, the AA in LBD and delirium involves a combination of HPA axis hyperactivity and a slight ACh downregulation. When the ACh deterioration is small, a larger AA insert of HPA axis dysfunction is necessary for the appearance of AA. On the other hand, when the ACh deterioration is large, a small AA insert of HPA axis dysfunction is sufficient. Of course, if the ACh deterioration is at a critical level, AA appears without HPA axis dysfunction. However, as previously discussed, if ACh is not deteriorated or overloaded, the intact ACh system can be upregulated and compensate for another AA insert. We refer to the conditions encompassed by this concept as “anticholinergic spectrum disorders” (Figure 5).

**Figure 6:**
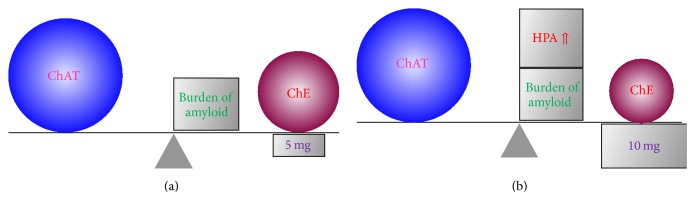
ChEI enhances ACh and enhanced ACh compensates for other AA inserts besides ACh downregulation. We prescribe three main ChEIs—donepezil, galantamine, and rivastigmine—to patients with mild or moderate stage AD. For donepezil, 5 mg doses are allowed in Japan (a). Of these ChEIs (including generic medicines), only Aricept is permitted for LBD patients in Japan (since September 2014). In general, the dose of Aricept is 10 mg for LBD (10 mg of donepezil is permitted only for severe AD patients in Japan) (b). We believe that the difference in required dosage between AD and LBD is because there is no other AA insert in AD besides ACh downregulation. However, another AA insert (e.g., HPA axis hyperactivity) is present in LBD, so it is important to upregulate ACh to compensate for this AA insert. AA: anticholinergic activity, ACh: acetylcholine, AD: Alzheimer's disease, ChEI: cholinesterase inhibitor, HPA axis: hypothalamic-pituitary-adrenal axis, and LBD: Lewy body disease. 5 mg (in (a)): 5 mg doses of donepezil; 10 mg (in (b)): 10 mg doses of Aricept (donepezil). This figure is reproduced from Konishi et al. [24] with the permission of Karger Publishers, Basel, Switzerland.

Possible pharmacotherapies for anticholinergic spectrum disorders are of course also important. Yilmaz et al. [32] described the case of a 19-year-old man with anticholinergic toxicity who complained of restlessness. Administration of 2 mg of physostigmine, which enhances ACh by acting as a cholinesterase inhibitor (ChEI), immediately alleviated the symptoms. This case report appears to confirm our previous view that when the anticholinergic toxicity is caused by an exogenous factor (e.g., prescribed medicine), the mechanism of AA appearance depends on the endogenous system that is directly related to the ACh downregulation. Indeed, Flacker and Lipsitz [7] and Plaschke et al. [8] also commented that AA is caused by endogenous factors. We speculate that prescribed medication, physical illness, and mental stress induce HPA axis hyperactivity, which causes AA in persons whose ACh system is downregulated or overloaded, such as the elderly and patients with AD or LBD. Therefore, we prescribe ChEI to patients with a suspected anticholinergic spectrum disorder. ChEI enhances ACh and enhanced ACh compensates for other AA inserts besides ACh downregulation. Three main ChEIs—donepezil, galantamine, and rivastigmine—are prescribed to patients with mild or moderate stage AD. In Japan, 5 mg doses of donepezil are allowed (Figure 6(a)). Of these ChEIs (including generic medicines), only Aricept® is permitted for LBD patients in Japan (since September 2014). In general, the dose of Aricept is 10 mg for LBD (10 mg of donepezil is permitted only for severe AD patients in Japan) (Figure 6(b)). We believe that the difference in required dosage between AD and LBD is because there is no AA insert in AD other than ACh downregulation, whereas another AA insert is involved in LBD, such as HPA axis hyperactivity. Therefore, it is important to upregulate ACh to compensate for this AA insert. As for delirium, we can confirm that ChEI exerts positive and sudden effects. ChEI should thus be prescribed to patients with these spectrum disorders to abolish AA by upregulating ACh. Indeed delirium is considered to be related to downregulation of ACh [33–35] and AA [36–38] and ChEIs are favorable for delirium [39–41]. Therefore, delirium is suggestive of “anticholinergic spectrum disorders.” We consider that this concept is enlarged to AD and LBD.

## Abbreviations

AA: Anticholinergic activity
ACh: Acetylcholine
AD: Alzheimer's disease
ChEI: Cholinesterase inhibitor
HPA axis: Hypothalamic-pituitary-adrenal axis
LBD: Lewy body disease
SAA: Serum anticholinergic activity.
